# Bilateral NAION and GPIbα gene

**DOI:** 10.1186/s12886-018-1010-0

**Published:** 2019-01-07

**Authors:** Christina S. Lim, Ajoy Sarkar, Christopher Knapp

**Affiliations:** 1 Ophthalmology Department, Lincoln County Hospital, Greetwell Road, Lincoln, Lincolnshire UK; 2 Department of Clinical Genetics, City Hospital Campus, The Gables, Gate 3, Hucknall Road, Nottingham, Nottinghamshire UK; 3 Present Address: Ophthalmology Department, Northampton General Hospital, Cliftonville Road, Northampton, UK

**Keywords:** Bilateral optic neuropathy, Platelet glycoprotein, GPIbα

## Abstract

**Background:**

It has been previously reported that one copy of the variable number tandem repeat (VNTR) B alleles of the GPIbα gene increases the risk of non-arteritic ischaemic optic neuropathy (NAION) and the second eye involvement. This is the first case where the presence of both alleles is associated with bilateral NAION.

**Case presentation:**

A 52-year-old male presented with loss of vision in one eye and was diagnosed with NAION. The following year, he suffered another attack of NAION in the fellow eye. Genetic testing showed that he had both copies of VNTR B alleles of the GPIbα gene.

**Conclusions:**

We report a case of bilateral NAION in the presence of two copies of VNTR B alleles of the GPIbα gene. This may have further implications for the function of platelet glycoproteins.

## Background

Non-arteritic ischaemic optic neuropathy (NAION) causes a severe irreversible sight loss. The incidence is estimated to be between 2.30–10.2 per 100,000 in the United States [1, 2]. NAION is associated with vascular risk factors such as hypertension, smoking, diabetes mellitus and hyperlipidaemia [3–8]. It is thought to be due to reduced perfusion by posterior ciliary arteries [9] although it is also suggested by Arnold that the lack of perfusion may be due to vasculopathy distal to the short posterior ciliary arteries affecting paraoptic branches or their tributaries within the disc, based on their detailed early filling fluorescein angiographic studies [10].

Platelets are an integral component of thrombus formation in an endothelial injury. The initial interaction between platelet and the vascular endothelium is mediated by the platelet glycoprotein (GP) complex [11]. The largest subunit of the complex is the GPIbα (OMIM# 606672) [12] which also has the binding domain for von Willebrand factor [3]. Polymorphisms in platelet glycoproteins have been found to be associated with increased risks of coronary artery disease and stroke [13–16]. There is also some evidence that aspirin may reduce the risk of second eye involvement in NAION [17, 18] but the evidence is equivocal [19, 20]. One study by Salomon et al showed that the presence of the variable number tandem repeat (VNTR) in the B allele of GPIbα increases the risk of NAION and second eye involvement [21]. Here we report a patient who presented with polymorphisms in both alleles of the GPIbα gene and suffered bilateral NAION.

## Case presentation

A 65-year-old male attended the eye clinic with a past history of sudden reduction of vision in the right eye (RE) when he was 52 years old, followed a year later by sudden reduction of vision in the left eye (LE). Extensive investigation in a tertiary referral centre had identified elevated homocysteine levels resulting from a gene mutation for the enzyme methylenetetrahydrofolate reductase (MTHFR) and hypercholesterolaemia. In the absence of other findings a diagnosis of bilateral NAION was made.

**Fig. 1:**
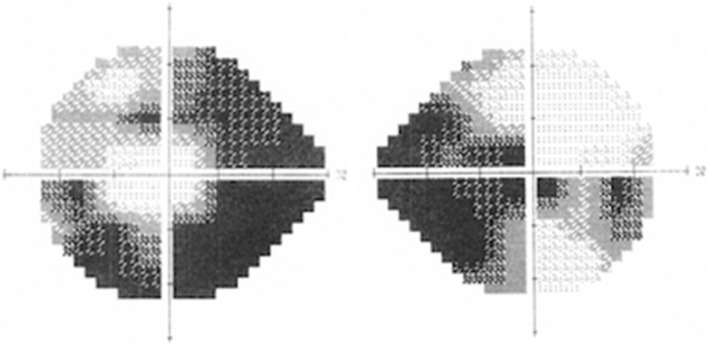
Automated Humphrey visual field 24-2 of both eyes

When seen in our clinic he had LogMar visual acuity of 0.2 in the RE and 0.0 in the LE. Colour vision was reduced in the right eye. Three out of 17 Ishihara test plates were correctly identified in the RE and 16 in the LE. Visual field testing (Humphrey 24–2) showed absolute superior and inferior nasal scotomas in the RE, and superior and inferior altitudinal scotomas with preservation of the central 20 degrees in the LE (Fig. 1). The optic nerves were pale with minimal cupping. In view of these unusual findings, further tests were arranged. A carotid ultrasound scan was normal as was an MRI scan of the optic nerves and brain. Referral to a clinical geneticist was arranged. The typical genetic mutations for Lebers herediary optic neuropathy and familial hypercholesterolaemia were not found, however a homozygous mutation in the GPIbα (VNTR B allele) was identified. The hyperlipidaemia and elevated homocysteine levels were managed by the endocrine team. Medication consisted of atorvastatin 40 mg ON, folic acid 400 mcg OD and clopidogrel 75 mg OD.

## Discussion and conclusions

Ischaemic optic neuropathy is caused by ischaemia of the anterior part of the optic nerve which is supplied by the short posterior ciliary arteries [9] although there was a suggestion that the origin of ischaemia may be distal to the short posterior ciliary arteries [10]. There are two types- arteritic optic neuropathy, caused by giant cell arteritis [7] and non-arteritic ischaemic optic neuropathy. The pathogenesis of the latter is unclear [8], however, a few risk factors have been identified such as hypertension, nocturnal arterial hypotension, diabetes mellitus, ischaemic heart disease, hyperlipidaemia and atherosclerosis [3–8].

Platelets play an important role in the coagulation in the event of an endothelial injury. This is mediated by platelet glycoprotein which also provides the binding site for von Willebrand factor. A study by Salomon, et al suggested that the presence of the VNTR B allele of GPIbα increases the risk of NAION with an odds ratio of 4.25 (95% CI = 1.67–10.82) and also second eye involvement [21]. Nine out of 16 patients with the allele (56.3%) had the second eye involvement whereas for those without, only 17 out of 72 patients (23.6%) had second eye involvement (*P* = 0.009) [20]. Polymorphisms in platelet glycoprotein have already been shown to increase the risk of thrombotic conditions such as ischaemic strokes [13, 14] and ischaemic heart disease [15, 16].

The platelet surface glycoproteins are divided into three groups I, II and III. The group I which is further divided into groups Ia, Ib Ic and GPIbα which is a surface membrane heterodimer with a larger α chain [22]. When there is an endothelial damage, platelets interact with the exposed subendothelial matrix via GP Ib-IX-V complex and the vWF factor [23]. The complex consists of one GPIbα subunit bonded to two molecules of GPIbβ via disulphide bonding which is then non-covalently bonded to GPIX and GPV [24]. The GPIbα of the complex provides the binding site to the immobilised vWF [24]. This interaction supports slow rolling of platelets and continuous adhesion [24] [25] under high shear stress [26]. It eventually leads to thrombus formation [27]. There are four variants of GPIbα which result from different numbers of VNTR. They are named A, B, C and D in the order of reducing number of repeats (4, 3, 2 or 1 repeat) and molecular mass [28]. There is little known in the literature regarding the VNTRs of GPIbα and its effect in thromboembolic disease. One study by Salomon et al suggested that the presence of the VNTR B allele of GPIbα was likely to increase platelet interaction with the endothelium hence leading to the occlusion of posterior ciliary arteries [21]. Here we report the first case of bilateral NAION in the presence of both VNTR B alleles of GPIbα. This case further supports the evidence that the VNTR B allele of GPIbα increases the risk of NAION including the second eye involvement. This may have further implications for the function of platelet glycoproteins.

## Abbreviations

GP: Glycoprotein
MTHFR: Methylenetetrahydrofolate reductase
NAION: Non-arteritic ischaemic optic neuropathy
VNTR: Variable number tandem repeat
